# Change of Programme

**Published:** 1868-02

**Authors:** 


*Change of Programme.*—The Goodyear Dental Vulcanite Company have notified the profession that all licenses expired on the first of January, 1868, and that they have decided, for the present year, to issue yearly office rights for certain sums, provided all previous dues are paid. So that instead of compelling Dentists to keep a special record and make quarterly returns, a certain sum is charged for a yearly office right, the company specifying the amount in each case, and increasing it according to their pleasure at the end of each year. They also threaten every practitioner with an injunction debarring him from all use of rubber, in case he refuses to comply with these terms. We refer our readers to the article *Interesting to Dentists*, in the present number of the Journal, and they can then decide with regard to the course they will pursue.

